# Unveiling the mechanism of remote epitaxy of crystalline semiconductors on 2D materials-coated substrates

**DOI:** 10.1186/s40580-023-00387-1

**Published:** 2023-08-30

**Authors:** Xuejing Wang, Joonghoon Choi, Jinkyoung Yoo, Young Joon Hong

**Affiliations:** 1 Center for Integrated Nanotechnologies, Los Alamos National Laboratory, Los Alamos, NM 87544 USA; 2 Department of Nanotechnology and Advanced Materials Engineering, GRI–TPC International Research Center, Sejong University, Seoul, 05006 South Korea

**Keywords:** Remote epitaxy, Epitaxy mechanism, Heterogeneous integration, Incommensurate materials, Two-dimensional materials, Semiconductors, Advanced manufacturing

## Abstract

Remote epitaxy has opened novel opportunities for advanced manufacturing and heterogeneous integration of two-dimensional (2D) materials and conventional (3D) materials. The lattice transparency as the fundamental principle of remote epitaxy has been studied and challenged by recent observations defying the concept. Understanding remote epitaxy requires an integrated approach of theoretical modeling and experimental validation at multi-scales because the phenomenon includes remote interactions of atoms across an atomically thin material and a few van der Waals gaps. The roles of atomically thin 2D material for the nucleation and growth of a 3D material have not been integrated into a framework of remote epitaxy research. Here, we summarize studies of remote epitaxy mechanisms with a comparison to other epitaxy techniques. In the end, we suggest the crucial topics of remote epitaxy research for basic science and applications.

**Graphical Abstract:**

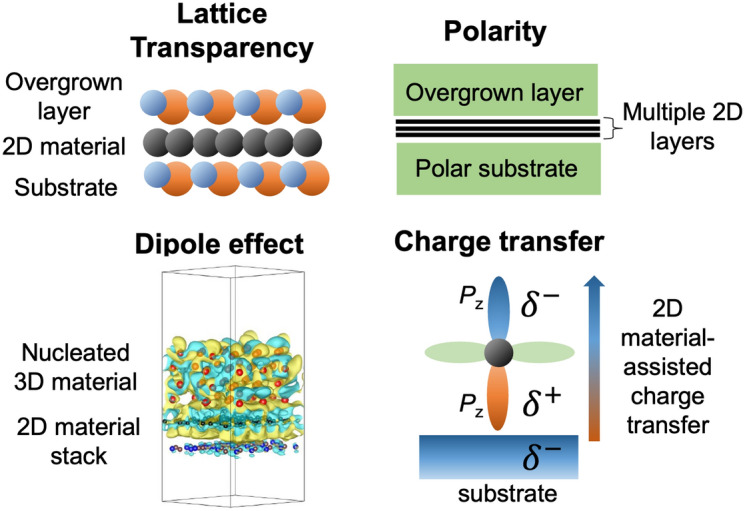

## Introduction

### Demands and challenges for next-generation electronics and optoelectronics

Advances in semiconductor epitaxy have catalyzed the development of various high-performance electronic and optoelectronic devices, including high electron mobility transistors (HEMTs), light emitting diodes (LEDs), laser diodes, photodetectors, etc. [1–4]. These developments have significantly impacted our daily lives by making them smarter, more compact, convenient, and sustainable. For next-generation semiconductor applications, technologies enabling high-performance, multi-functional, and free-form devices are essential. Moore’s law highlights the scaling challenges faced by nano- and micro-devices in the current semiconductor industry [5, 6]. High-density heterogeneous integration of devices could overcome difficulties in achieving high-performance and multi-function devices beyond Moore [7, 8]. Additionally, novel designs such as three-dimensional integration and foldable or wearable electronics pose immediate hurdles over traditional solid-state thin film templates [7, 9]. The challenges could be addressed by developing new innovative methods in epitaxy and subsequent device fabrication process [10].

Two-dimensional (2D) materials have emerged as a promising solution due to their weak inter-planar van der Waals (vdW) forces, allowing for the physical separation of individual layers without surface dangling bonds [11]. These ultrathin nanomaterials have already been integrated into multi-functional vertical heterostructures by stacking them over again and applied to flexible electronic devices [12–15]. However, it is still challenging to prepare single crystalline 2D materials that can meet the stringent requirements of microelectronics standards [16]. Moreover, numerous defects (e.g., wrinkles, tears, folds, etc.) are unintentionally formed on the ultrathin nanolayers during the transfer and stacking process, [17, 18] complicating their use in standard microelectronics processing. Meanwhile, conventional epitaxial thin films have been chemically, thermally, and mechanically “peeled off” by chemical etchants [19], pulsed lasers [20], and mechanical spalling, [19] respectively, to create free-standing membranes, like 2D materials. The membranes were either diced-and-assembled or bonded-and-patterned to integrate the devices on a template, presenting the native outstanding physical properties of the epitaxial membranes [21–24]. In addition, exceptional structural durability was obtained after the diced devices were assembled on flexible templates [25]. The membrane-based approaches demonstrated their distinct potential for addressing the challenges toward high-performance, hetero-integration, and flexible device applications [10]. Nonetheless, concerns related to the scalability of 2D materials, difficulties in high-density assembly for hetero-integration, and potential damage to epi-film surfaces from chemical etching or laser-assisted melting may affect high-density hetero-integration and the overall performance of the nanodevices.

### Opportunities offered by 2D materials as substrate

The ultimate goal for next-generation electronics/optoelectronics research is to develop reliable methods for preparing large-scale, high-quality epitaxial semiconductor membranes that can be easily “lifted off” and “stacked on” for hetero-integration and flexible devices [8]. This approach will enable to create high-performance/multi-functional electronic and optoelectronic devices, satisfying the demands of various applications, including wearable electronics, foldable devices, and advanced integration technologies. Developing such methods requires breakthroughs in material synthesis, transfer techniques, and device fabrication processes, which must leverage the unique properties of 2D materials and advanced epitaxy techniques [11, 26–29]. One potential solution is the direct growth of thin films on top of 2D layered materials [30–32]. Chung et al. reported on growing epitaxial GaN layers on ZnO nanowalls-buffered graphene, becoming the earliest success in achieving epitaxial semiconductor films on graphene [30]. Due to the difficulty in forming a continuous film on graphene, oxygen-plasma treatment is carried out on graphene, enhancing its chemical activity by creating step-edges that facilitate the formation of chemical bonds with the overlayer. The natural growth of high-density ZnO nanowalls on these step-edges plays a critical role in realizing the heteroepitaxial growth of GaN on graphene without forming polycrystals or unwanted islands. This work demonstrates that it is possible to create high-quality thin film epilayers by engineering the surface of 2D material and to stably operate the resulting epilayer LEDs after freely transferring them to the desired surface. This can be regarded as the original work on the releasable epitaxy, and it has significant implications in offering diverse and appropriate applications guidance in the subsequent research areas of vdW and remote epitaxy. Moreover, recent advances in preparation of single crystalline 2D materials, on which nucleation of conventional materials is not significantly governed by defects on 2D materials, have provided other opportunities of understanding the roles of 2D materials as substrates and manufacturing of high-quality releasable membranes of conventional materials [33, 34].

Since the 2D materials have no dangling bonds on their surfaces, energy minimization through the formation of chemical bonds does not drive epitaxial growth. As a result, an unconventional and novel epitaxy regime has emerged: the concept of “vdW epitaxy” was firstly introduced by Koma et al. in 1984 [35], which signifies epitaxial growth capable of accommodating significant lattice mismatches at the substrate/overlayer interface by virtue of the vdW gap, free of surface dangling bonds [36, 37]. The vdW epitaxy comprehensively encompasses specific types of epitaxial structures, including heteroepitaxy of purely 2D stacks, 2D materials grown on conventional wafers, and thin film nucleation on top of 2D materials. Among numerous 2D materials, graphene, a honeycomb-bonded monoatomic-thick sheet of carbon, has emerged as the most favorable 2D candidate for the vdW epitaxy substrate due to excellent thermal and mechanical stability and thickness-controlled synthesis. Significantly, graphene is the thinnest material and possesses no permanent dipole, making it an ideal candidate for investigating the interacting forces involved in the unconventional epitaxies discussed in this review article.

Intriguingly, graphene exhibits “transparency” from multiple perspectives: it is optically transparent (< 2% absorption) at 1–2 monolayer thickness [38], making it an ideal 2D transparent conductor; it also demonstrates wetting transparency [39], meaning that the wetting behavior of the underlying substrate remains unaffected by the thin graphene on top; “lattice transparency” is a relatively new concept that has emerged in recent years, serving as the fundamental basis for remote epitaxy, a novel phenomenon in material systems that include atomically thin 2D materials for creating a gap. This review summarizes the unique aspects of remote epitaxy, compared to other epitaxy schemes, such as conventional epitaxy limited by materials compatibility, nanoepitaxy, and vdW epitaxy. The current understanding of the remote epitaxy mechanism based on polarity-governed lattice transparency will be discussed. Subsequently, the challenges of lattice transparency as the key mechanism will also be discussed with the recent results. The review of remote epitaxy will lead us to the perspectives of remote epitaxy research to achieve a thorough understanding of the mechanism and to realize applications in advanced manufacturing and heterogeneous integration.

## General categories of epitaxy

### Conventional epitaxy: homoepitaxy, strained epitaxy, and domain matching epitaxy

Conventional epitaxy broadly refers to epilayer nucleation and growth on substrate by forming covalent or ionic bonds at the interface through physical or chemical vapor deposition (CVD) methods, such as pulsed laser deposition (PLD), molecular beam epitaxy (MBE), metal–organic vapor phase epitaxy (MOCVD), hydride vapor phase epitaxy (HVPE), etc. [1, 40]. A wide range of materials, including elemental semiconductors, compound semiconductors (e.g., II–VI, III–V, carbides, etc.), ceramic oxides, perovskites, or others, can be grown using well-established recipes that yield single crystalline, smooth surface coverage suitable for practical device applications. Critical factors for obtaining high-quality epilayers and heterostructures include the selection of appropriate substrates with similar crystal structure, thermal expansion coefficient, and careful control of growth conditions.

**Fig. 1:**
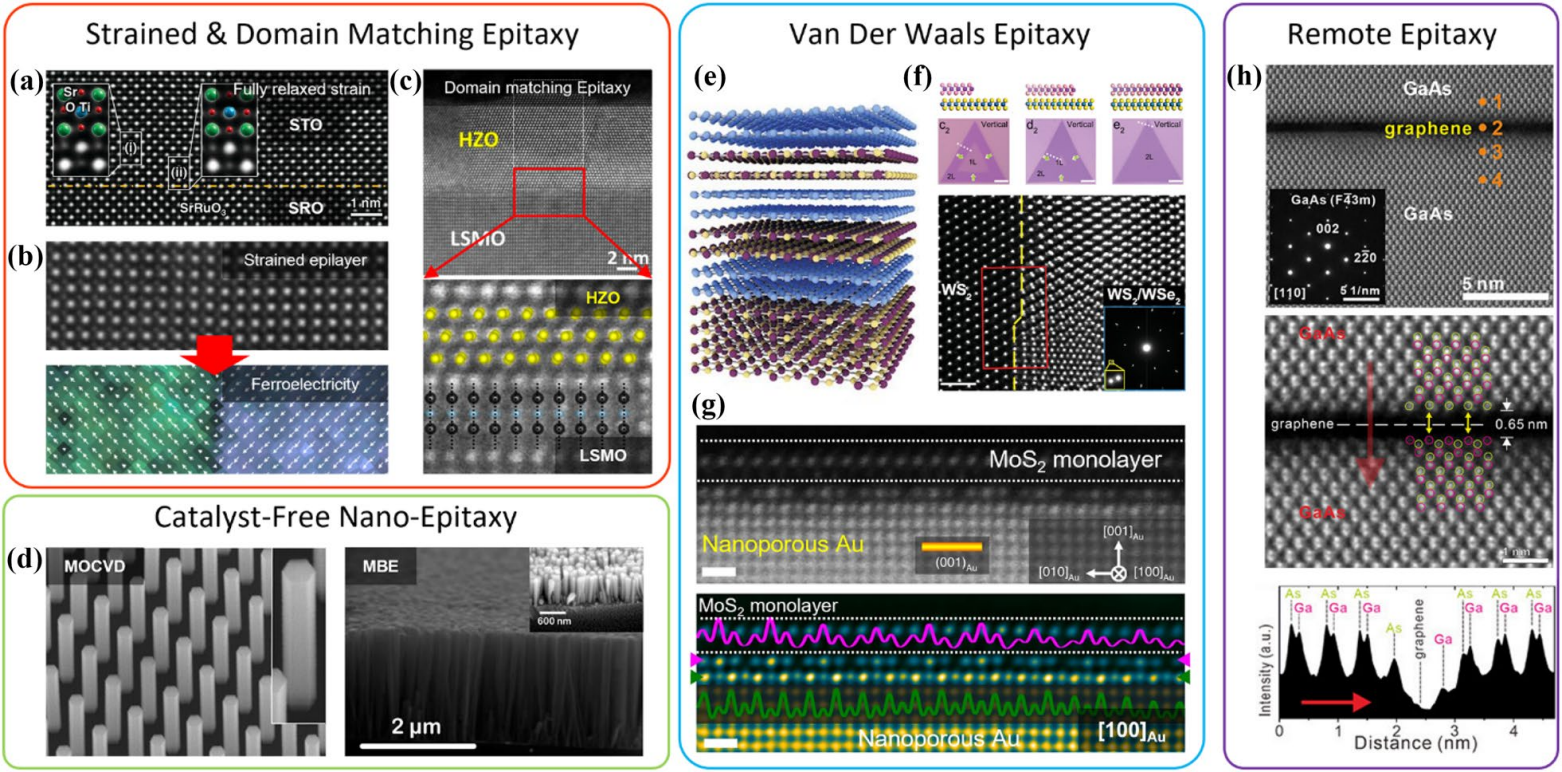
Types of Epitaxy. Conventional thin film heteroepitaxy with strained or domain matching lattice relationship. **a** Fully relaxed SrTiO_3_(STO) thin film epitaxy on SrRuO_3_(SRO) buffered STO substrate. **b** Domain Wall interface and its displacement vector map of BiFeO_3_ grown on STO substrate. **c** ZnO doped HfO_2_ (HZO) on La_0.7_Sr_0.3_MnO_3_(LSMO) domain matching epitaxy with a 9:10 matching relationship. **d** Catalyst-free nano-epitaxy of semiconductor NWs using metal–organic chemical vapor deposition (MOCVD) and plasma-assisted molecular beam epitaxy (PA-MBE). SEM tilted view of InGaAs NW arrays on patterned substrates (left) and cross-sectional SEM image of InGaN NWs grown by on (001) Si (right). **e** Heteroepitaxy composed of purely 2D layered materials. **f** MoS_2_/WSe_2_ heterostructures with increase of W/Se composition ratio (left to right) and its HRSTEM micrograph at the WS_2_/WSe_2_ heterointerface. **g** Raw HAADF-STEM image and and false color viewed HAADF-STEM iamge of monolayer MoS_2_ grown on nanoporous gold substrate from the [98]_Au_. **h** HRSTEM micrographs and atomic line profile of remote epitaxial GaAs/monolayer graphene/GaAs heterointerface

Generally, there are several distinct modes or schemes in the conventional epitaxy. (1) Homoepitaxy or commensurate epitaxy, where the film/substrate interface is strain-free and/or composed of the same material, is the most intuitive epitaxy mode and will not be discussed in detail. For example, SrRuO_3_ (SRO) is considered as an ideal buffer conductive layer on SrTiO_3_ (STO) due to their comparable lattice constants (Fig. 1a) [41]. (2) Heteroepitaxy, where strained growth can be maintained up to a certain thickness before fully relaxed. A strained epilayer can exhibit unexpected physical properties such as ferroelectricity (Fig. 1b) [42]. (3) Domain matching epitaxy, a special case with the M:N lattice matching relationship between the epilayer and substrate, typically generates periodic misfit dislocations to minimize the strain or structural disorder for the epitaxial growth. This growth scheme enables the combination of material systems from different space groups, for example, the Zr doped HfO_2_ (HZO) and La_0.7_Sr_0.3_MnO_3_, as shown in Fig. 1c [43].

Indeed, single crystalline substrates, such as Si, GaAs, sapphire, and other single crystalline oxides, are commonly used for epitaxy due to commercial reasons of cost, large-area process, well-established legacy process, etc. For this reason, most semiconductor epitaxy is performed through heteroepitaxy [44], where strain control is always a significant challenge. The thickness of the wafer is much greater than that of epilayer, making strain control in the epilayer difficult without forming strain-compliant or strain-controlled layers (e.g., buffer, porous epitaxial layer, superlattices) [45–50]. For functional devices, composition-modulated thin heterostructures, such as quantum wells, are necessary with precisely controlled thickness and homogeneous composition. However, the accurate control in the strained epilayer is not easily accomplishable. For instance, indium segregation is observed in indium-rich InGaN-based red LEDs [51, 52], despite the complete compositional solubility of indium in InGaN [53]. The limited choice of commercial wafers makes developing high-quality devices with controlled strain in a designed structure challenging. Regarding the epilayer separation, the chemical and laser lift-off is usually used, which leaves chemical and physical damage to the heterostructures in the epitaxial overlayer. Thus, the damages induced from the aforementioned lift-off and dicing procedures could substantially degrade the device performances, where the feature size of devices becomes smaller for high-density integration [54–58]. Therefore, the epitaxy methods discussed in the following sub-sections offer potential alternatives for relaxing or controlling strain and damage-less lift-off and dicing of epilayer, which could lead to improved device performances and the development of next-generation electronic and optoelectronic devices.

### Nanoepitaxy

Nanoepitaxy, introduced here as a distinct type of epitaxy, involves the growth of epi-structures in contact with the substrate on a sub-micrometer scale. Self-assembled quantum dots (QDs) via Stranski–Krastanov (SK) growth mode are the first examples of nanoepitaxy. Shape and nucleation of extended defects in a QD are governed by balance between adatom adhesive force and surface adhesive force during SK growth. Another notable example, for which extended defect formation can be minimized, is the growth of single-crystalline nanowires (NWs) on crystalline substrates. One of the earliest examples is the growth of crystalline Si NWs using the Au-catalyzed vapor–liquid–solid (VLS) method [59]. The VLS NW growth has been extensively studied, and it has been shown to produce axial heterostructures with graded composition modulation along NW length. The VLS occurs due to a metal catalyst droplet, which segregates the alloyed composition to the top of the NW during the growth process [60–62]. The metal droplet acts as a nucleation site for the NW material, allowing it to grow in a preferred direction while the alloyed components are concentrated towards the top of the NW for segregation. However, metal catalyst-based NWs growth is not solely governed by VLS mechanism. For instance, growth of GaN NWs by MBE is governed by a combination of self-catalyst of Ga droplets and screw dislocation-driven mechanism [63, 64].

Catalyst-free nanoepitaxy has emerged as a preferable approach for growing semiconductor NWs without the inevitable metal catalyst contamination. This method presents abrupt clean interfaces for both axial and radial heterostructures. Several catalyst-free nanoepitaxy mechanisms have been proposed, including oxide-assisted growth (OAG), self-catalytic VLS, dislocation-driven growth, and surface energy minimization. For the OAG method, deposited Si sub-oxide clusters act as nuclei to facilitate the formation of Si NW in a preferred orientation. Oxygen atoms in Si sub-oxide are expelled from Si during the growth and diffuse to the edge, forming SiO_x_ shell that prevents further radial expansion of the NW. The OAG nanoepitaxy with preferred crystallographic orientation is also affected by dislocations in the growth direction, with Si {111} facets exposure to minimize surface energy. The self-catalytic VLS of NW, as explained by Mohammad et al., is driven by the formation and retention of liquid droplets from constituent metal, adsorption and incorporation of vapor-phase species into liquid droplets, and the creation of nucleation sites for NW growth [65, 66]. Surface energy minimization of NWs represents another principal mechanism of nanoepitaxy. Non-centrosymmetric wurtzite semiconductors, such as GaN and ZnO, tend to elongate along c-axis to minimize the area of unstable *c*-planes [67]. The spontaneous nucleation and subsequent elongation along a preferred crystallographic orientation have motivated selective-area nanoepitaxy for preparing NW arrays with predetermined positions and precisely controlled diameters and heights. This control also allows the growth of quantum well structures and position-controlled light-emitting architectures on crystalline substrates (e.g., Si, sapphire, GaAs, etc.) [68–74]. These catalyst-free nanoepitaxy have been successfully demonstrated by both MOCVD and MBE techniques for monolithic integration of III–V NWs for both electronic and optoelectronic device applications (Fig. 1d) [75, 76]. More importantly, the NW morphology offers an opportunity of releasing the strain caused at the heterostructure interface through the large surface area of the NW periphery.

Research areas of nanoepitaxy also include selective area epitaxy (SAE) based on various mechanisms, such as contrast of precursor adsorption and desorption on chemically and physically patterned substrates. SAE has been considered as localized epitaxy of thin films at desired locations. Recent progress in nanomaterials growth has demonstrated that SAE can be useful to control morphology and sizes of nanostructures [77, 78]. Studies of SAE have brought fruitful discussions on chemical/physical patterning of surfaces of substrates to deliver contrast of adsorption and desorption of precursor molecules and adatoms rather than a growth mechanism.

### Epitaxy on 2D materials: vdW epitaxy and remote epitaxy

As briefly discussed in the Introduction, 2D materials offer opportunities to investigate the interactions between the grown layer and substrate, which are physically separated by the vdW gap. When vdW materials are introduced as substrates, conventional growth and nucleation mechanisms are not applicable due to the absence of chemical bonds (i.e., covalent and ionic bonds) across the vdW gap [79]. To utilize a 2D material, an underlying substrate is required to support the ultrathin 2D layer. In a 2D/substrate structure, mixed interacting forces from the 2D material and underlying substrate influence the adatom of the epilayer during the growth. Depending on the relative strength of these interacting forces, epitaxy without chemical bonds is classified as either vdW or remote epitaxy. Various materials platforms for comparing the interactions across the vdW gap have been realized through either vdW or remote epitaxy. In this section, we will provide more details about these intriguing growth schemes that enable the hetero-integration of epitaxial devices and flexible designs, representing many new aspects of materials chemistry/physics and processing.

**Fig. 2:**
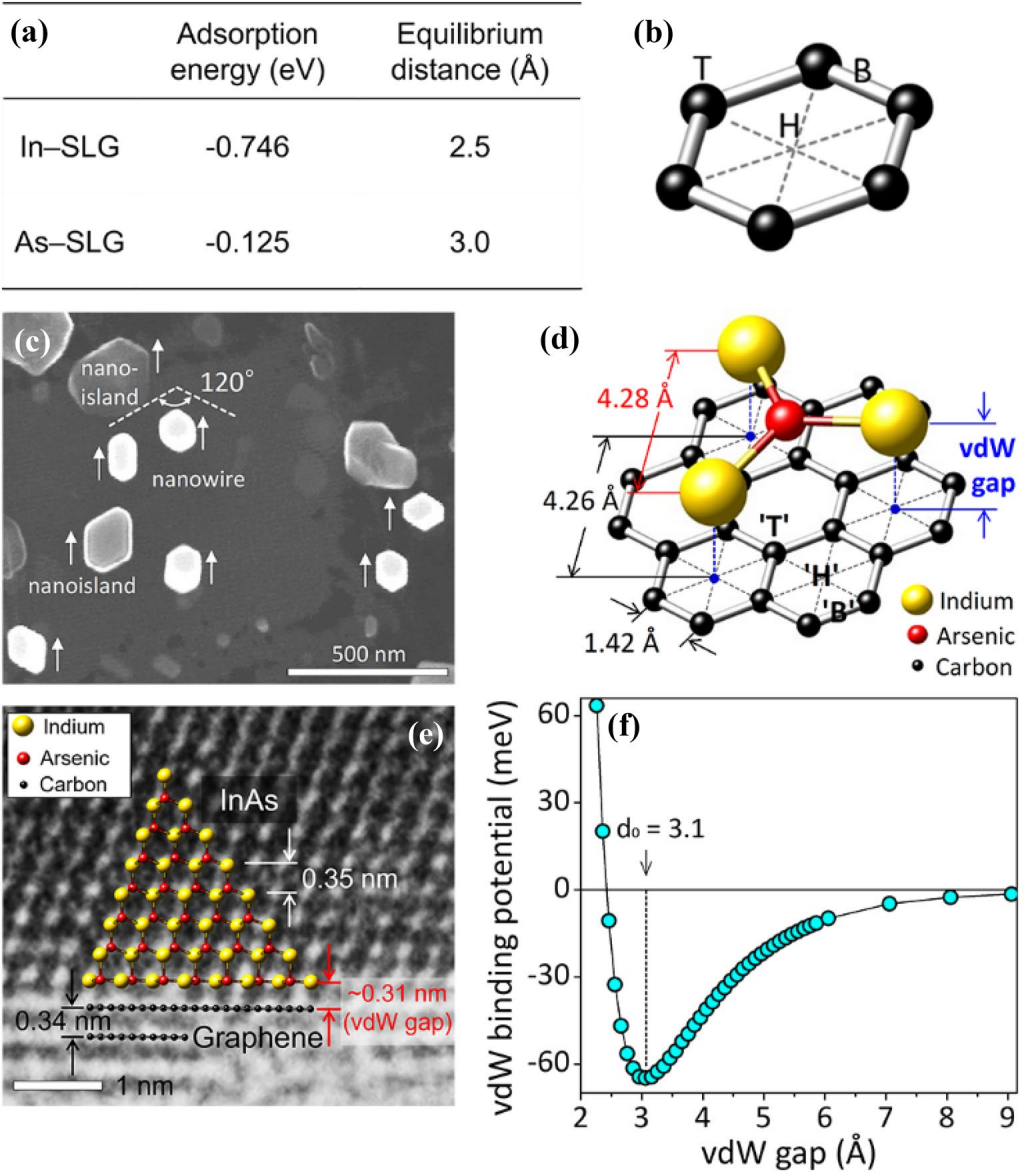
vdW epitaxy of InAs NWs on graphene. **a** Adsorption energy and equilibrium distance of indium and arsenic adatom on graphene. **b** Possible adatom sites on graphene, noted with H, T, and B, standing for hollow, top, and bridge, respectively. **c** Top-view SEM image of InAs NWs grown on suspended graphene. **d** The ball-and-stick model for vdW InAs/graphene epitaxial structure. **e** HRTEM image of the cross-sectioned InAs/graphene. **f** vdW binding energy of InAs on graphene, plotted as a function of vdW gap, simulated by first-principles calculation

When the vdW force from 2D material is more dominant than that from the underlying substrate, the vdW force governs the adatom migration, nucleation, and initial epilayer growth at the earliest epitaxy stage. The process is the so-called “vdW epitaxy” [80] and an epitaxial relationship exists between the epilayer and 2D material. The vdW epitaxial relation is clearly observed by growing semiconductors on 2D/SiO_2_ and suspended 2D layers [81, 82]. For the vdW epitaxy, the initial growth is of particular interest with respect to adatom and nucleation. Hong et al. grew InAs NWs on suspended graphene through MOCVD, resulting in a uniform in-plane alignment of vertical hexagonal NWs, as shown in Fig. 2c. The vdW epitaxial InAs/graphene system is simulated through first-principles calculations, revealing that indium atoms exhibit greater adsorption energy on graphene than arsenic (Fig. 2a) [82] The hexagonal hollow (‘H’, Fig. 2b) was identified as the most stable site for adsorption on graphene, rather than carbon bridge (‘B’) and carbon top (‘T’), according to the calculation work by Chan et al. [83] Following the initial indium adatom, arsenic participates in forming InAs nuclei, as depicted in Fig. 2d. Subsequently, InAs NWs grow exclusively along < 111 > B (Fig. 2e). The calculated vdW adsorption energy for the InAs/graphene system is 64 meV with an equilibrium vdW gap of 3.1 Å (Fig. 2f), which is in good agreement with TEM observation.

When the interacting force from the underlying substrate is stronger than the vdW force from the top 2D layer, the crystalline substrate remotely dictates the crystallographic registration (i.e., crystal symmetry and orientation) of the epilayer through 2D material. It is called “remote epitaxy” [84, 85]. Such long-range force through the 2D layer is discussed with regard to polarity formed by bonding ionicity [86]. In the remote epitaxy, the crystal (or domain) orientation of 2D materials does not impact the remote epitaxial relationship [87]. However, not all uses of 2D layer/crystal substrate can guarantee the remote epitaxy: the 2D layer must be ultrathin enough to allow the attracting force from the substrate to penetrate, and the maximum thickness of the 2D layer for the remote epitaxy depends on the bonding ionicity of the underlying substrate [86].

The vdW epitaxy includes various novel epitaxial structures, such as 2D on 2D (Fig. 1e) [13], 2D on 3D [88] and 3D on 2D [82]. In this review, description of dimensionality is based on density of states not on shapes. Therefore, majority of the prepared materials on 2D materials can be considered as 3D materials unless physical properties of low-dimensional structures is given. Examples shown in Fig. 1f are CVD-grown vertical WS_2_/WSe_2_ epitaxial heterostructure with sharp atomic interface in between [89]; the vdW epitaxy can also be achieved on a bulk substrate, e.g., monolayer MoS_2_ on nanoporous Au, shown in Fig. 1g [90]. The vdW epitaxy of 3D on 2D has generally been studied for compound semiconductors (e.g., InAs on graphene) [91]. It is noteworthy that the parallel progress in advanced microscopy and data processing for visualizing atomically sharp cross-sections and moiré superlattices of these heterostructures provides valuable insights into understanding the mechanisms of vdW epitaxy. The epitaxy of 3D on 2D and 2D on 3D are referred to as quasi-vdW epitaxy due to the presence of dangling bonds on the 3D material. Whether actual chemical bonding occurs has intrigued many researchers in the field. In principle, no strong interaction sharing or donating electrons via dangling bonds on the vdW substrate mediates the formation of an epilayer, even for the quasi-vdW epitaxy. Thus, 2D on 3D or 3D on 2D epitaxy has shown structurally decoupled interfaces with a large gap of at least two or three Å, whose distance is much greater than the length of typical chemical bonds (i.e., covalent, ionic, and metallic bonds). Since the dangling bonds on the 3D side have no direct chemical bonds with the 2D substrate, the vdW substrate enables domain-aligned growth of highly incommensurate materials without forming threading dislocations through the decoupled interface. In this review article, we generally refer as the vdW epitaxy, without making a distinction between it and quasi-vdW epitaxy.

Remote epitaxy represents another aspect of material interactions across two vdW gaps, on and beneath the 2D material. Regardless of the orientation or domain of 2D material, the epilayer’s crystal symmetry and orientation follow that of the substrate. For instance, Fig. 1h displays a GaAs epilayer grown on a graphene-coated GaAs substrate, revealing that diatomic Ga–As dumbbells are perfectly aligned across a 0.65 nm gap [92]. Additionally, remote epitaxially grown GaN on graphene/*c*-Al_2_O_3_ showed the same epitaxial relation of GaN grown on *c*-Al_2_O_3_. Thus, the strength of remote epitaxy lies in utilizing conventional large-scale crystalline wafers. Furthermore, despite the large lattice mismatch, the high-quality epilayer can be achievable where the remote epitaxy is implemented. The high-quality epilayers prepared by remote epitaxy provide various opportunities of advanced device manufacturing and realization of heterostructures composed of incommensurate materials due to their quality and releasable structures [93].

The slippery vdW surface of 2D materials efficiently releases the strain and does not form the dislocation as well. The weak attraction between the epilayer and wafer across the 2D layer enables easy peel-off of the epilayer through metal stressor or thermal release tape-assisted delamination method. As for the 2D layer, graphene has become the most favorable candidate, primarily due to its thermal stability and compatibility with the high-temperature conditions required for growing epilayers. The “lattice transparency” characteristic, which is the key element enabling remote epitaxy, will be discussed in the next section.

The motivation for growing high-quality semiconductor films includes three aspects: (1) fundamental explorations in understanding vdW interactions or the mechanisms of remote epitaxy; (2) releasing these functional membranes for stacking the into vertical or lateral heterostructures, while enabling substrate reuse, an attractive feature for commercializing the method toward industrial applications; (3) utilizing membranes or epilayers to develop flexible optoelectronic devices and wearable sensors or chips. In the following sections, we will discuss the governing mechanisms that enable these remote epitaxy schemes, as well as recent progress in developing flexible devices.

## Governing mechanisms and factors of remote epitaxy

### 2D/3D substrate: role of dangling bonds on 3D

**Fig. 3:**
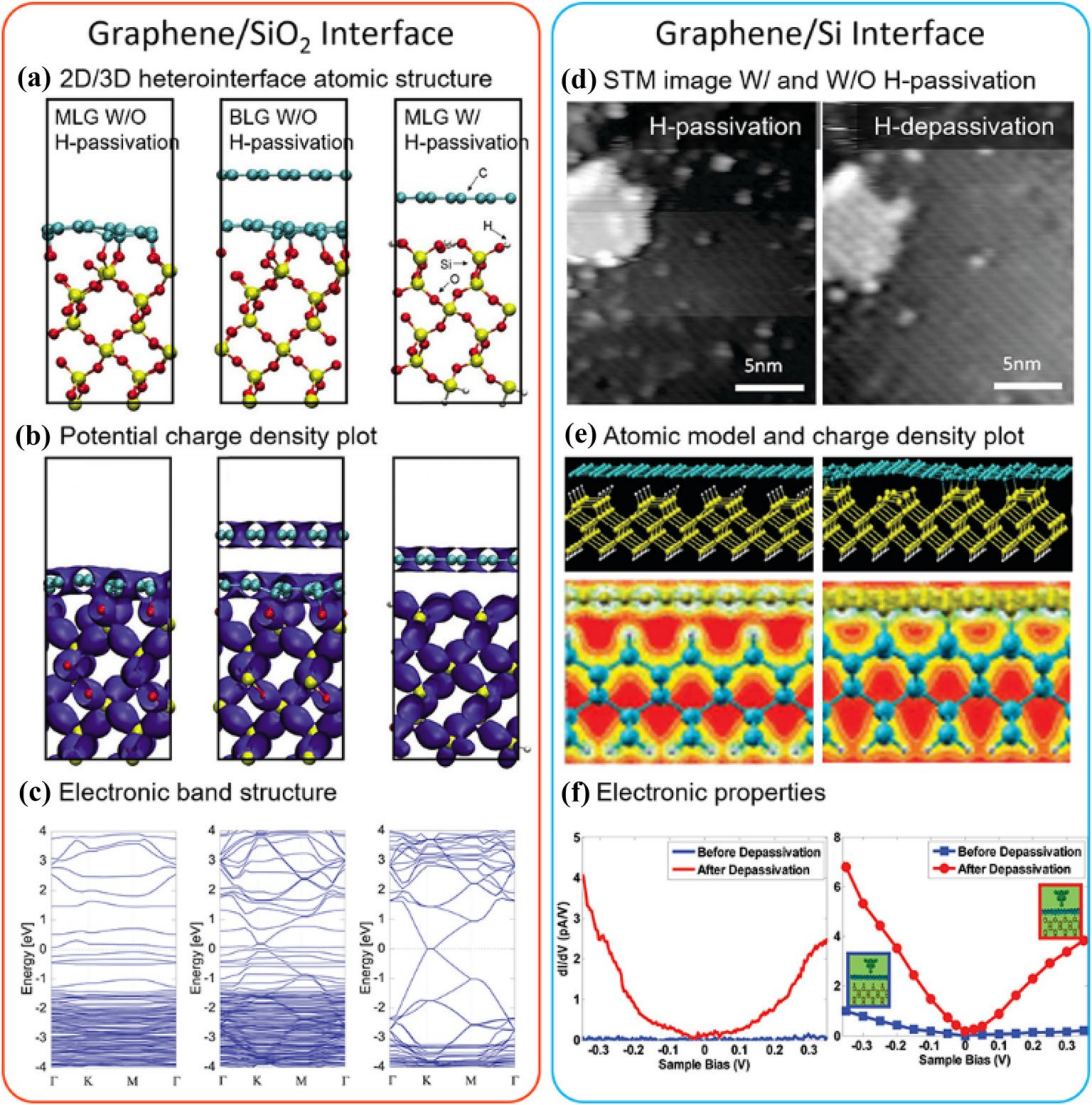
Surface interactions. DFT calculations of potential interactions at graphene/SiO_2_ (crystalline, O-terminated) substrate interface for three cases: monolayer graphene, bilayer graphene, monolayer graphene on H-passivated SiO_2_ substrate in the red box. **a** atomic models, **b** charge density plots, **c** band structures. Graphene/Si (001) substrate interface in the blue box. **d** STM images before and after H-depassivation. **e** Atomic models and charge density distribution of graphene on Si with H-passivated and H-depassivated Si surface. **f** Experimental and simulated tunneling dI/dV versus sample bias, respectively

The first question to address is whether there are any interactions, in terms of atoms, electrons, or phonons at the 2D/3D interface, that modify electronic properties or surface chemistry. Over the years, numerous studies have confirmed the existence of such interactions and the possibility of manipulation through chemical treatments. For example, Shemella et al. [94] theoretically unveiled interactions between single-layer (1L)- and double-layer (2L)-graphene with the O-terminated crystalline SiO_2_ surface using density functional theory (DFT), indicating that carbon atoms tend to form C–O covalent contacts for graphene placed on natural SiO_2_ surfaces, but this is limited only to 1L graphene. There are no such interactions for hydrogen(H)-passivated SiO_2_ surfaces (Fig. 3a). The predicted potential charge density plot at these interfaces with a vdW gap indicates charge transfer between 1L graphene and native SiO_2_ surfaces (Fig. 3b), and the electronic band structure shows a switch from semiconducting to semi-metallic properties when no interaction occurs between graphene and H-passivated SiO_2_ surfaces (Fig. 3c). Notably, DFT consistently provides reliable predictions and insights for experimental explorations.

Later in 2010, Lyding’s findings [95] on semi-lattice-transparent graphene on GaAs substrate, resulting from its electronic effect, brought significant interest to the unique “lattice transparency” property of graphene in altering structural properties of the substrate material. These findings align with another work reported by Aluru et al. in [96], which also provided experimental observations of visualizing such transparent substrate structures through graphene after the H-depassivation process. Figure 3d displays scanning tunneling microscopy (STM) images of the graphene lattice on top of H-passivated and H-depassivated Si(100), respectively. Similarly, the H-passivation protects the substrate surface from chemical interaction with carbon atoms, showing a non-lattice-transparent graphene layer with respect to the Si surface, while the depassivation process breaks the bonds between H–C, returning to the original clean Si surface where potential covalent bonding occurs. The predicted atomic model and the charge density plots (Fig. 3e) using DFT calculations corroborate the experimental results. Furthermore, the electronic properties show consistency between measured and transport conductivity, indicating a transition to metallic nature after depassivation (Fig. 3f), consistent with Shemella’s prediction of the band structure.

**Fig. 4:**
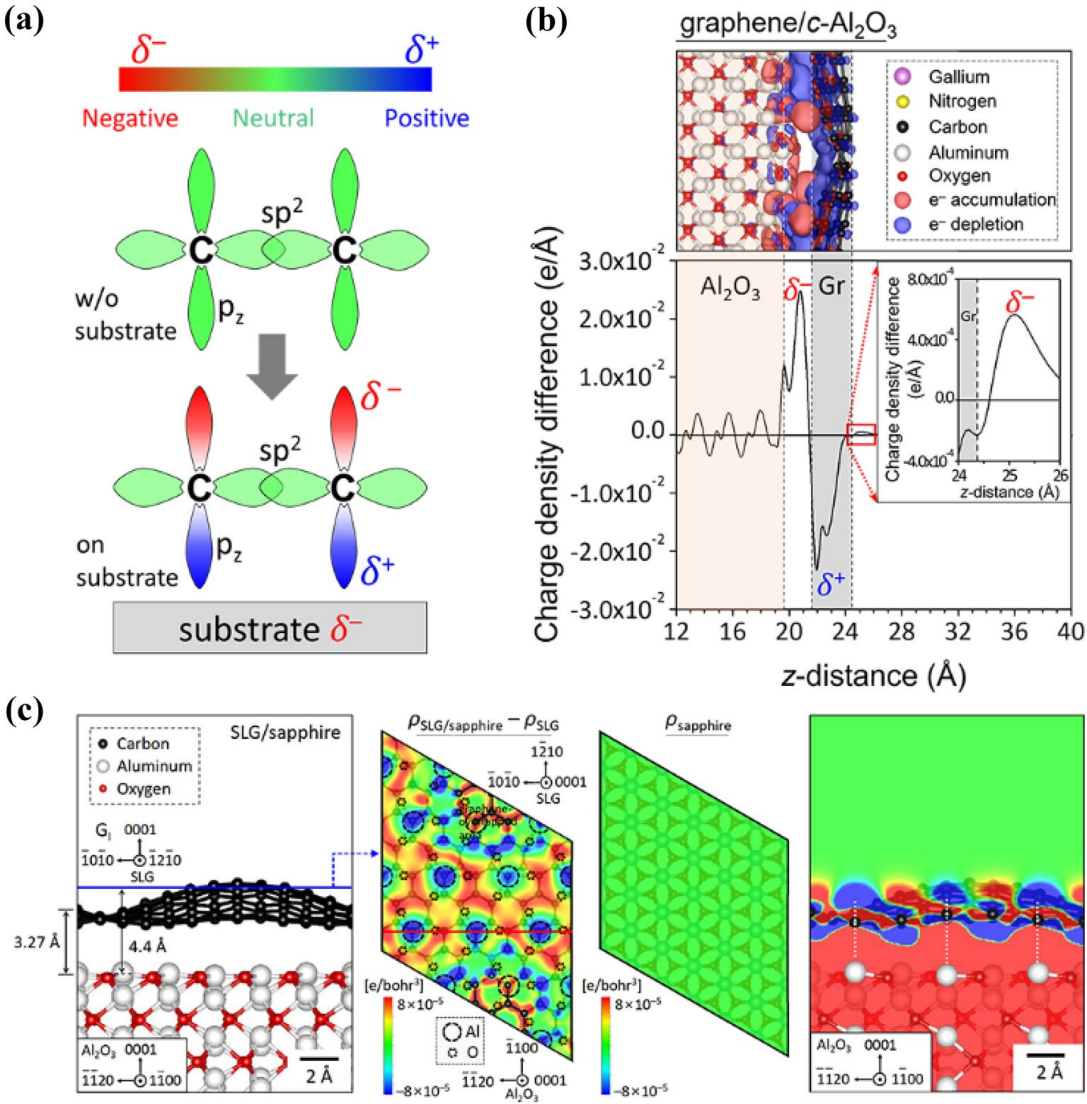
Principle of charge transfer and redistribution through graphene layer. **a** Schematics of charge transfer through *p*_z_ orbitals of graphene layer affected by substrate net charge. The amount of net electron charge was relatively denoted by blue–green–red color column for electron deficiency, neutral, and electron accumulation state in colors ordered. **b** Atomic model, electron charge density mapping, and charge density difference plot as a function of z-distance at 1L graphene/c-Al_2_O_3_ system. **c** Atomic structure and electron charge density maps on 1L graphene/c-Al_2_O_3_ system

Carbon atoms in graphene have two *p*_z_ orbitals perpendicular to the graphene plane, and these diametrically symmetric orbitals play a crucial role in remote epitaxy. When a negative local charge on a crystal substrate reaches the bottom of graphene, the downward *p*_z_ orbital becomes positively charged because there are no electron sharing or donations and the orbitals merely overlap (Fig. 4a). Consequently, the upward *p*_z_ orbital turns negative to maintain charge neutrality in graphene. In this manner, the *p*_z_ orbitals mediate to transfer the charge as like induced dipole, and the surface charge patterns of the underlying crystal substrate are replicated to the graphene surface (Fig. 4b, c). For example, every carbon atom placed on aluminum atoms shows a positive charge density difference (CDD, blue in Fig. 4c) in 1L graphene/c-Al_2_O_3_ substrate, and without graphene the CDD maps at the same height surface show a neutral charge. This suggests the ability of graphene to transfer local charge, as depicted in Fig. 4c. However, this charge pattern replication is attenuated as increasing graphene thickness. Hence, there exists the maximum graphene thickness for remote epitaxy, as reported by Kong et al. [86].

### Lattice transparency of 2D layer: a key mechanism of remote epitaxy

**Fig. 5:**
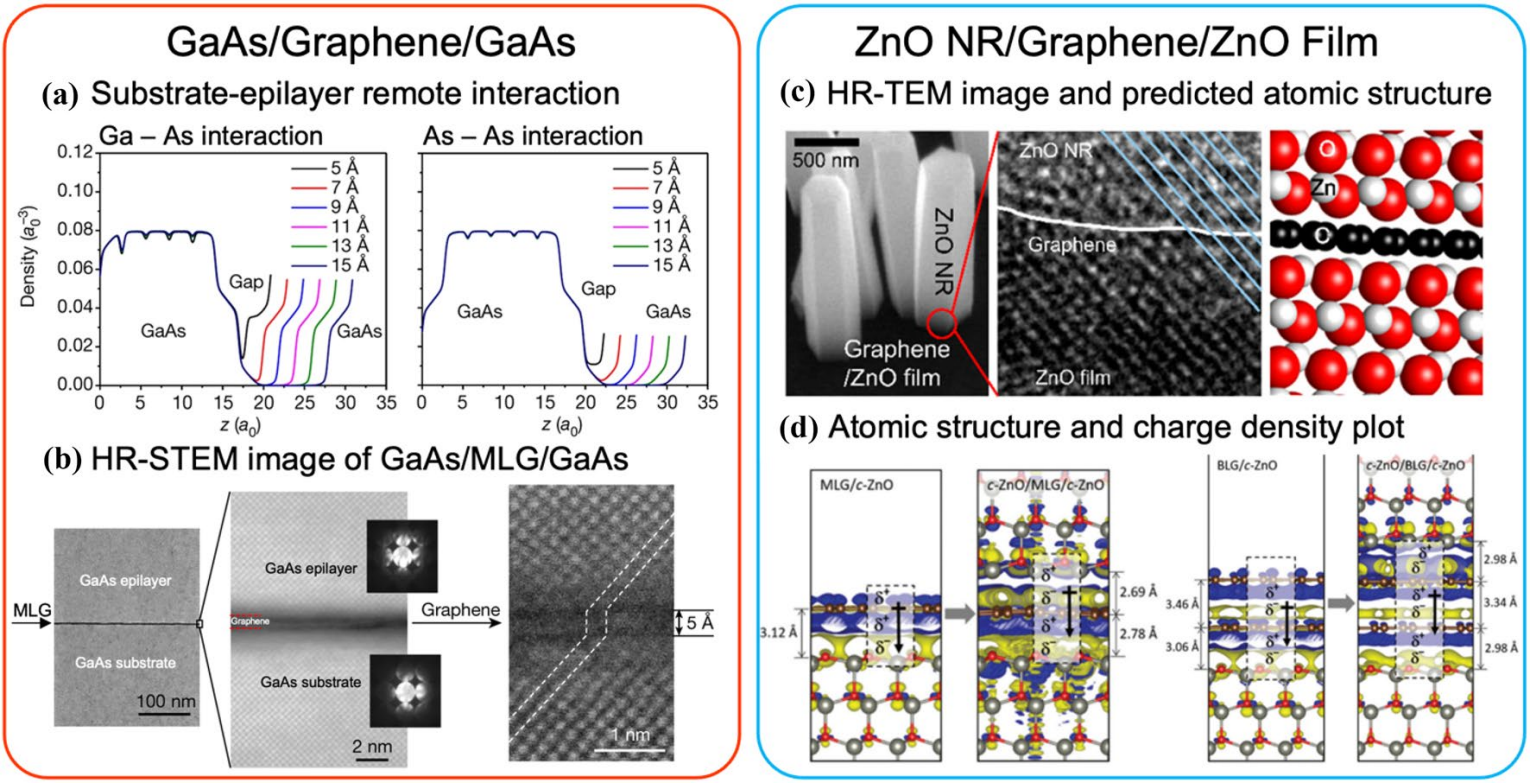
Remote epiaxy through lattice transparent vdWs layer. Remote homoepitaxy of GaAs/graphene/GaAs in the red box. **a** DFT calculated average electron density along the GaAs slabs for As-Ga interaction and As-As interaction, respectively. Significant charge density exists inside the separated gap of about 9 Å. **b** STEM micrographs at the GaAs/graphene/GaAs heterostructure interface with a sharp interface with thickness of 5 Å. Remote homoepitaxy of ZnO nanorods/graphene/ZnO film in the blue box. **c** SEM tilted view of the ZnO nanorods grown on graphene/ZnO substrate and its HRTEM image across the interface with predicted atomic structure. **d** Atomic structure and charge density difference of remote homoepitaxial c-ZnO/MLG/c-ZnO and c-ZnO/BLG/c-ZnO heterointerfaces

**Fig. 6:**
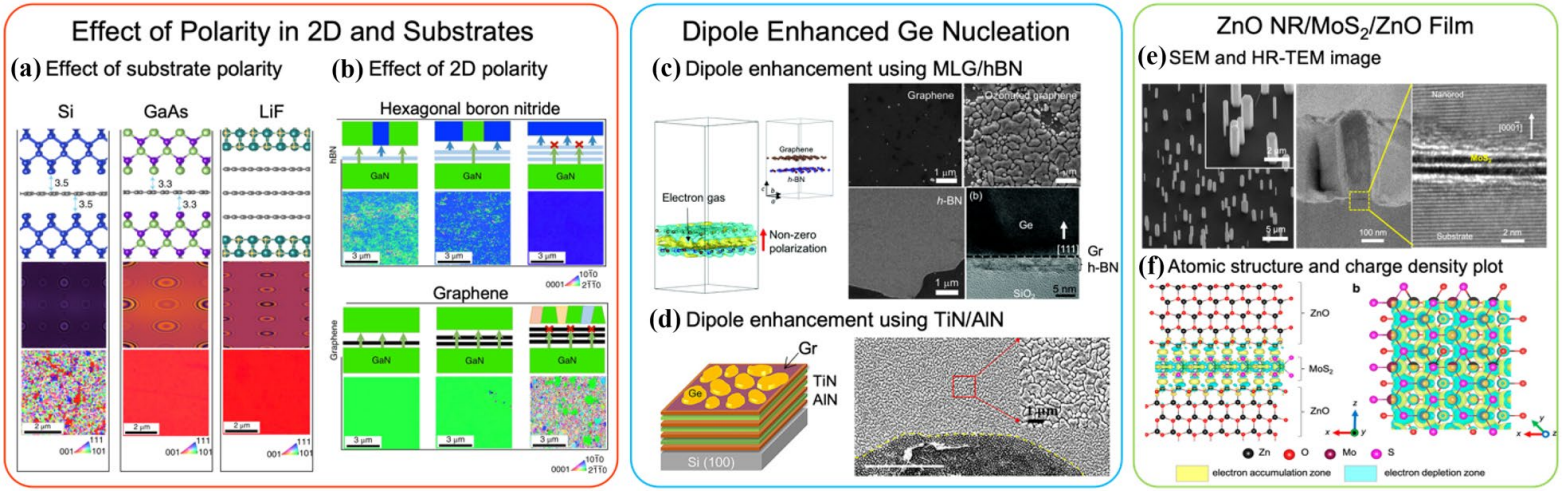
Polarity effect of substrate polarity and in 2-D materials in the red box. **a** From top to bottom: atomic model, DFT simulated potential fluctuation (meV) map, and the EBSD mapping of the crystalline orientation for Si/1L-graphene/Si (left column), GaAs/1L-graphene/GaAs (middle coloum), and LiF/3L-graphene/LiF (right column), respectively. **b** Schematics of the polarity effect of 2-D materials and corresponding EBSD mapping for GaN remote homoepitaxy with h-BN and graphene, respectively. Enhancing Ge thin film nucleation through dipole moment from 2D and substrate in the blue box. **c** 3D DFT simulation of electron density in the graphene/h-BN stack, red arrow denoting the direction of non-zero polarization. SEM images of Ge nucleation on top of graphene, ozone-treated graphene, and h-BN on SiO_2_/Si substrate and HR-TEM micrograph for Ge grown on graphene/h-BN stacked SiO_2_/Si substrate. **d** 3D illustration of Ge nucleation on graphene/TiN-AlN hyperbolic metamaterial stack and corresponding SEM image of the Ge surface. **e** Tilted SEM, low-magnification BF-TEM image and HRTEM at the ZnO/1L-MoS_2_/ZnO interface. **f** DFT calculated charge density distribution from both side and top projections

The examples presented in Fig. 6 show clues of the lattice transparency from the substrate surface through graphene, even though the underlying Si substrate has no bonding ionicity. Two critical factors can be identified for the lattice transparency: (1) the number of 2D layers that can be tolerated and (2) surface termination of ionic bonded atoms in the underlying crystal. Kim et al. [84] provided insightful pathways to these factors. The calculated charge density profile suggests that 2L graphene can be tolerated for As-terminated and Ga-initiated slabs, while only 1L graphene for As–As intersections (Fig. 5a). The actual growth confirms the single-crystalline quality of GaAs grown on 1L graphene/GaAs(001) substrate, while polycrystalline for 2L and 3L graphene/GaAs substrates. High-angle annular dark field (HAADF) scanning transmission electron microscope (STEM) reveals a high-quality interface, with GaAs atomic columns perfectly aligned remotely through the 5 Å gap (Fig. 5b). Recent work by Kim et al. [92] directly shows that As-initiated GaAs was formed on 1L graphene/Ga-terminated GaAs substrate, suggesting that no polarity inversion occurred through the graphene gap. More interestingly, the authors demonstrate that GaAs(001), InP(001), and GaP(001) epilayers, which are III–V compounds with bonding ionicity, can be formed through remote epitaxy, and exfoliated using metal stressor and thermal release tape, for preparing LED devices with performance comparable to the LEDs prepared by conventional epitaxy [84]. As the first report on remote epitaxy, this study opens up tremendous possibilities for understanding the remote epitaxy mechanism structurally and applying it commercially to a wide range of optoelectronic devices for heterogeneous integration and preparing flexible devices using this growth scheme.

Lattice transparency of graphene in realizing ZnO nanorod remote homoepitaxy has also been demonstrated [97], showing atomic column alignment as resolved by high-resolution transmission electron microscopy (HRTEM) (Fig. 5c). Calculation of interfacial binding energy provides insights into the remote epitaxy mechanism. The binding energy for direct homoepitaxy of ZnO is always high, while it decreases when graphene is inserted. Intuitively, the binding energy for ZnO remote epitaxy is higher for growth on graphene/crystalline Zn-terminated ZnO (*c*-znt-ZnO) as compared to the nucleation on graphene/disordered Zn-terminated ZnO (do-znt-ZnO). It is meaningful that the charge density distribution at these interfaces is calculated to understand how graphene electronically interacts with the substrate and thus affects the as-grown epilayer. Figure 5d shows that the existence of graphene enhances charge redistribution and shortens equilibrium distance, which means graphene acquires the “ZnO-mimic” charge distribution from the underneath ZnO substrate, reinforcing the interactions with epi-ZnO growth. Briefly, lattice transparency is an important factor in realizing remote epitaxy. Such transparency is closely correlated with charge redistributions across the vdW gap to enhance the binding energy for the epilayer.

### Polarity: a factor governing available material combinations for remote epitaxy

DFT calculations can predict the number of graphene layers or other 2D materials that can be inserted to achieve a single-crystalline epilayer. In the other work [86], Kong et al. discussed that polarity, which arises from the materials’ bonding ionicity or atomic bonding characteristics, could affect the epilayer crystallinity,. Such ionicity can vary for different materials (both 2D and 3D) and the thickness of the 2D interlayer. Examples displayed in Fig. 6a clearly demonstrate the single crystallinity of GaAs remote homoepitaxy through 1L graphene, while electron backscatter diffraction (EBSD) mapping reveals polycrystalline epilayers for Si/1L graphene/Si and GaN/3L graphene/GaN [86]. Similar to the binding energy calculation mentioned earlier, calculating the potential fluctuation of the 2D/substrate surface predicts the likelihood of potential energy being transferred through the 1L graphene. For example, the well-transmitted potential of GaAs or LiF leads to remote epitaxial growth, while covalently bonded Si presents significant attenuation of the potential field, resulting in a polycrystalline Si epilayer regardless of graphene thickness. With the increase in ionicity, more layers of graphene can be inserted without screening out the potential field, as observed from the single-crystalline LiF film grown across 3L graphene.

Polarity exists in both vdW 2D materials and crystalline substrates; even for hexagonal boron nitride (h-BN), polarity still exists, although covalent bonding interactions are dominant in h-BN. To achieve successful remote epitaxy, predictions can be made based on charge distribution and binding energy calculations, as previously mentioned, while also considering materials’ ionicity and thickness of 2D insertion to design and expand remote epitaxy to a broader range of material systems. Specifically, for the remote homoepitaxy of GaN, as shown in Fig. 6b, [86] when grown on top of h-BN layers, polarity must be considered for both 2D and substrate layers. GaN epilayers are expected to be grown as polycrystalline on thinner h-BN due to a mixture of potential fields caused by the polarity of both h-BN and GaN, while GaN forms single-crystalline film on thicker h-BN because dominant vdW attracting force from h-BN drives the vdW epitaxy of GaN on h-BN [98]. The polarity from substrate could be smeared out throughout the 2D layer. This suggests that additional factors need to be considered when the vdW interlayer has its own polarity, which could be beneficial but more complicated to be applied to either remote or vdW epitaxy. For the use of graphene, the situation is simpler since the covalently bonded carbon atoms do not contribute to polarity, but for more than 3L-graphene, such remote interaction might be too weak to penetrate.

### Beyond lattice transparency: out-of-plane dipole moment, interference, attenuative charge transfer

Studying remote epitaxy involves addressing two questions: (1) nucleation on a 2D material and (2) the role of the 2D material as a substrate. The postulation of ‘lattice transparency’ alone is insufficient to develop a model for remote epitaxy because an ideal 2D material has no surface dangling bonds and steps that facilitate the nucleation of a 3D material of overlayer. In most cases, adsorption coefficients of precursors or adatoms of 3D materials on 2D materials, especially graphene, are too low to form sufficiently dense nuclei to form a continuous thin film. The nucleation has been explained by surface energy modification through formation of out-of-plane dipole moment.

A 2D material is not an inert component in a substrate architecture consisting of a crystalline 3D substrate and the 2D material for remote epitaxy, as charge transfer occurs between the substrate and the 2D layer. In a recent report by Yoo et al. [99] stacking 1L graphene with 1L h-BN significantly enhances the nucleation of Ge, which does not effectively occur either on 1L graphene or on h-BN alone. The underlying mechanism involves the induced out-of-plane dipole moment in the graphene/h-BN stack. Charge transfer between graphene and h-BN leads to the accumulation of electrons along the vdW gap between h-BN and graphene. The accumulated charges generate an out-of-plane dipole moment, which increases the surface energy of the topmost graphene layer (Fig. 6c). Engineering the out-of-plane dipole moment in a 2D material to facilitate the nucleation of 3D materials on the 2D layer has been demonstrated through multiple approaches: for example, graphene placed onto polarized ferroelectric thin film and metal/dielectric (TiN/AlN) multi-layers generates inherent out-of-plane dipole moments (Fig. 6d) [100]. Although still in its early stages, the dipole engineering can be considered a universal strategy for nucleating 3D materials on various 2D materials.

The out-of-plane dipole engineering brings insight into the roles of 2D materials and their stacks with other 2D and 3D materials as substrates for remote epitaxy, going beyond lattice transparency. Recent studies demonstrate that such transparency can be extended to monolayer TMDs, such as 1L-MoS_2_. Figure 6e–f illustrate the remote epitaxy of ZnO nanorods/1L-MoS_2_/ZnO, which can be achieved on both ZnO nanorods and films [101]. The DFT-calculated charge density distribution indicates a more energetically favorable O-terminated ZnO alignment across MoS_2_, i.e., the O–Zn//S–Mo–S//Zn–O along the out-of-plane direction, which also induces a polarity inversion considering the S–Zn bonding interactions. The colored electron accumulation (Fig. 6f) also suggests charge transfer between MoS_2_ and ZnO layers, appearing to have a similar driving mechanism to remote epitaxy through graphene. The polarity inversion implies that the monolayer MoS_2_ interferes with the penetration of the surface potential of the underlying ZnO.

Another recent study of the epitaxy of AlN on graphene/SiC provides an insight of the mechanism of remote interaction through graphene [102]. The analysis of charge distribution in the AlN/graphene/SiC system shows that the graphene constructs the remote interaction between the SiC substrate and the crystalline AlN overgrown layer through mediation of attenuative charge transfer. The study indicates that there are plenty of room to explore mechanism of remote epitaxy besides lattice transparency.

## Outlooks

Remote epitaxy, a nascent research field, shows a typical pathway of progress in science. The underlying principles of remote epitaxy have been expanded from the simple and clear explanation of ‘lattice transparency’ to the incorporation of various ideas to explore opportunities for heterogeneous integration. Diverse observations have made discussions in the field fruitful. Nevertheless, there are still questions on the mechanisms of remote epitaxy, directly related to the validation of the concept of ‘remote epitaxy’. Therefore, the following questions are critical to the future of remote epitaxy research in basic science.

We need to explore the boundaries of the materials domain in which remote epitaxy is available. The current framework to understand remote epitaxy is based on lattice transparency and field penetration of the substrate through a 2D layer. Therefore, remote epitaxy has been studied with non-centrosymmetric 3D material on graphene. However, remote epitaxy on a non-graphene (MoS_2_) layer and the formation of cubic phases of 3D materials on graphene have been reported. The experimental observations indicate that remote epitaxy can be applicable for the growth of centrosymmetric 3D materials on non-graphene 2D materials. Pushing the limit of remote epitaxy can bring novel opportunities for understanding heterogeneous integration across the vdW gap and device manufacturing with existing infrastructure.

Remote epitaxy studies still lack a decisive visualization of penetration of the potential of the substrate through a 2D layer, although remote epitaxy is founded on a hypothesis of lattice transparency. Visualization of atom structures of Si underneath monolayer graphene was accomplished by STM. Nevertheless, the STM technique is not applicable to the realistic conditions of remote epitaxy. An in situ*/in operando* characterization technique to probe the atom arrangement of a 3D material on or beneath a 2D material will be a key method for understanding remote epitaxy in detail. Modeling at multi-scales encompassing atom scale and mesoscale is also necessary to obtain a fundamental understanding of remote epitaxy. Integrated theory suite, including quantum mechanics and vdW interaction in multi-hetero-layers, of which sizes are up to several tens-nanometers, will be a versatile tool.

Materials design to realize novel and unprecedented functionalities is being entered into the realm of remote epitaxy research as the available materials grown by remote epitaxy have been expanded. Stacked crystalline membranes of incommensurate materials with atomically sharp interfaces and heterostructures composed of non-graphene 2D materials and semiconductors are notable examples of recent accomplishments of remote epitaxy. Remote epitaxy research is expanding to the realization of novel devices besides economical manufacturing, which has been a key application. Unconventional material combinations not limited by material compatibility will open opportunities for novel devices. However, the devices require suitable designs for utilization.

The three topics discussed above are intertwined and require the integration of multi-discipline. Remote epitaxy as a key hub for interdisciplinary collaboration will advance our understanding of nanoscience and broadly impacts basic science and applied research.

## Data Availability

Not applicable.

## References

[1] Herman MA, Richter W, Sitter H (2013). Epitaxy: Physical principles and technical implementation.

[2] Dora Y, Chakraborty A, McCarthy L, Keller S, DenBaars SP, Mishra UK (2006). IEEE Electron Device Lett..

[3] Nakamura S (1998). Annu. Rev. Mater. Sci..

[4] Jung M, Gaddam V, Jeon S (2022). Nano Convergence.

[5] Akinwande D, Huyghebaert C, Wang C-H, Serna MI, Goossens S, Li L-J, Wong HSP, Koppens FHL (2019). Nature.

[6] Wang SY, Liu XX, Zhou P (2022). Adv. Mater..

[7] Liu Y, Huang Y, Duan X (2019). Nature.

[8] Kum H, Lee D, Kong W, Kim H, Park Y, Kim Y, Baek Y, Bae S-H, Lee K, Kim J (2019). Nat. Electron..

[9] Choi C, Kim H, Kang J-H, Song M-K, Yeon H, Chang CS, Suh JM, Shin J, Lu K, Park B-I, Kim Y, Lee HE, Lee D, Lee J, Jang I, Pang S, Ryu K, Bae S-H, Nie Y, Kum HS, Park M-C, Lee S, Kim H-J, Wu H, Lin P, Kim J (2022). Nat. Electron..

[10] Kim H, Chang CS, Lee S, Jiang J, Jeong J, Park M, Meng Y, Ji J, Kwon Y, Sun X, Kong W, Kum HS, Bae S-H, Lee K, Hong YJ, Shi J, Kim J (2022). Nat. Rev. Methods Primers.

[11] Kim J, Park H, Hannon JB, Bedell SW, Fogel K, Sadana DK, Dimitrakopoulos C (2013). Science.

[12] Geim AK, Grigorieva IV (2013). Nature.

[13] Novoselov KS, Mishchenko A, Carvalho A, Castro Neto AH (2016). Science.

[14] Choi M, Bae S-R, Hu L, Hoang AT, Kim SY, Ahn J-H (2020). Sci. Adv..

[15] Ahn J-H, Hong BH (2014). Nat. Nanotechnol..

[16] Hao Y, Bharathi MS, Wang L, Liu Y, Chen H, Nie S, Wang X, Chou H, Tan C, Fallahazad B, Ramanarayan H, Magnuson CW, Tutuc E, Yakobson BI, McCarty KF, Zhang Y-W, Kim P, Hone J, Colombo L, Ruoff RS (2013). Science.

[17] Ullah S, Yang X, Ta HQ, Hasan M, Bachmatiuk A, Tokarska K, Trzebicka B, Fu L, Rummeli MH (2021). Nano Res..

[18] Jang B, Kim C-H, Choi ST, Kim K-S, Kim K-S, Lee H-J, Cho S, Ahn J-H, Kim J-H (2017). 2D Mater..

[19] Cheng C-W, Shiu K-T, Li N, Han S-J, Shi L, Sadana DK (2013). Nat. Commun..

[20] Kelly MK, Vaudo RP, Phanse VM, Gorgens L, Ambacher O, Stutzmann M (1999). Jpn. J. Appl. Phys..

[21] Kang C-M, Lee J-Y, Kong D-J, Shim J-P, Kim S, Mun S-H, Choi S-Y, Park M-D, Kim J, Lee D-S (2018). ACS Photonics.

[22] Ahn J-H, Kim H-S, Lee KJ, Jeon S, Kang SJ, Sun Y, Nuzzo RG, Rogers JA (2006). Science.

[23] Park S-I, Xiong Y, Kim R-H, Elvikis P, Meitl M, Kim D-H, Wu J, Yoon J, Yu C-J, Liu Z, Huang Y, Hwang K-C, Ferreira P, Li X, Choquette K, Rogers JA (2009). Science.

[24] Viventi J, Kim D-H, Vigeland L, Frechette ES, Blanco JA, Kim Y-S, Avrin AE, Tiruvadi VR, Hwang S-W, Vanleer AC, Wulsin DF, Davis K, Gelber CE, Palmer L, Van der Spiegel J, Wu J, Xiao J, Huang Y, Contreras D, Rogers JA, Litt B (2011). Nat. Neurosci..

[25] Yoon J, Jo S, Chun IS, Jung I, Kim H-S, Meitl M, Menard E, Li X, Coleman JJ, Paik U, Rogers JA (2010). Nature.

[26] Shim J, Bae S-H, Kong W, Lee D, Qiao K, Nezich D, Park YJ, Zhao R, Sundaram S, Li X, Yeon H, Choi C, Kum H, Yue R, Zhou G, Ou Y, Lee K, Moodera J, Zhao X, Ahn J-H, Hinkle C, Ougazzaden A, Kim J (2018). Science.

[27] Kim Y, Suh JM, Shin J, Liu Y, Yeon H, Qiao K, Kum HS, Kim C, Lee HE, Choi C, Kim H, Lee D, Lee J, Kang J-H, Park B-I, Kang S, Kim J, Kim S, Perozek JA, Wang K, Park Y, Kishen K, Kong L, Palacios T, Park J, Park M-C, Kim H-J, Lee YS, Lee K, Bae S-H, Kong W, Han J, Kim J (2022). Science.

[28] Kim H, Liu Y, Lu K, Chang CS, Sung D, Akl M, Qiao K, Kim KS, Park B-I, Zhu M, Suh JM, Kim J, Jeong J, Baek Y, Ji YJ, Kang S, Lee S, Han NM, Kim C, Choi C, Zhang X, Choi H-K, Zhang Y, Wang H, Kong L, Afeefah NN, Ansari MNM, Park J, Lee K, Yeom GY, Kim S, Hwang J, Kong J, Bae S-H, Shi Y, Hong S, Kong W, Kim J (2023). Nat. Nanotechnol..

[29] Shin J, Kim H, Sundaram S, Jeong J, Park B-I, Chang CS, Choi J, Kim T, Saravanapavanantham M, Lu K, Kim S, Suh JM, Kim KS, Song M-K, Liu Y, Qiao K, Kim JH, Kim Y, Kang J-H, Kim J, Lee D, Lee J, Kim JS, Lee HE, Yeon H, Kum HS, Bae S-H, Bulovic V, Yu KJ, Lee K, Chung K, Hong YJ, Ougazzaden A, Kim J (2023). Nature.

[30] Chung K, Lee CH, Yi GC (2010). Science.

[31] Chung K, In Park S, Baek H, Chung J-S, Yi G-C (2012). NPG Asia Mater..

[32] Chung K, Oh H, Jo J, Lee K, Kim M, Yi G-C (2017). NPG Asia Mater..

[33] Jang H-S, Lim J-Y, Kang S-G, Seo Y-M, Moon J-Y, Lee J-H, Whang D (2020). ACS Nano.

[34] Kim KS, Lee D, Chang CS, Seo S, Hu Y, Cha S, Kim H, Shin J, Lee J-H, Lee S, Kim JS, Kim KH, Suh JM, Meng Y, Park B-I, Lee J-H, Park H-S, Kum HS, Jo M-H, Yeom GY, Cho K, Park J-H, Bae S-H, Kim J (2023). Nature.

[35] Koma A, Sunouchi K, Miyajima T (1984). Microelctron. Eng..

[36] Utama MIB, Peng Z, Chen R, Peng B, Xu X, Dong Y, Wong LM, Wang S, Sun H, Xiong Q (2011). Nano Lett..

[37] Hong YJ, Lee C-H (2015). in Semiconductors and Semimetals.

[38] Bae S, Kim H, Lee Y, Xu X, Park J-S, Zheng Y, Balakrishnan J, Lei T, Ri H, Song KYI, Kim Y-J, Kim KS, Özyilmaz B, Ahn J-H, Hong BH, Iijima S (2010). Nat. Nanotechnol..

[39] Rafiee J, Mi X, Gullapalli H, Thomas AV, Yavari F, Shi Y, Ajayan PM, Koratkar NA (2012). Nat. Mater..

[40] Stringfellow GB (1982). Rep. Prog. Phys..

[41] Lee D, Lu H, Gu Y, Choi S-Y, Li S-D, Ryu S, Paudel TR, Song K, Mikheev E, Lee S, Stemmer S, Tenne DA, Oh SH, Tsymbal EY, Wu X, Chen L-Q, Gruverman A, Eom CB (2015). Science.

[42] Nelson CT, Vasudevan RK, Zhang X, Ziatdinov M, Eliseev EA, Takeuchi I, Morozovska AN, Kalinin SV (2020). Nat. Commun..

[43] Estandía S, Dix N, Chisholm MF, Fina I, Sánchez F (2020). Cryst. Growth Des..

[44] Ayers JE, Kujofsa T, Rago P, Raphael J (2016). Heteroepitaxy of semiconductors: theory, growth, and characterization.

[45] Mynbaeva M, Titkov A, Kryganovskii A, Ratnikov V, Mynbaev K, Huhtinen H, Laiho R, Dmitriev V (2000). Appl. Phys. Lett..

[46] Fitzgerald EA, Xie YH, Monroe D, Silverman PJ, Kuo JM, Kortan AR, Thiel FA, Weir BE (1992). J. Vac. Sci. Technol. B.

[47] Hiramatsu K, Akasaki TD (1993). Jpn. J. Appl. Phys..

[48] Beanland R, Dunstan DJ, Goodhew PJ (1996). Adv. Phys..

[49] Brault J, Gendry M, Grenet G, Hollinger G, Desieres Y, Benyattou T (1998). Appl. Phys. Lett..

[50] Kandaswamy PK, Bougerol C, Jalabert D, Ruterana P, Monroy E (2009). J. Appl. Phys..

[51] Meng YL, Wang LS, Zhao GJ, Li FZ, Li HJ, Yang SY, Wang ZG (2018). Phys. Status Solidi A.

[52] Lin YS, Ma KJ, Hsu C, Feng SW, Cheng YC, Liao CC, Yang CC, Chou CC, Lee CM, Chyi JI (2000). Appl. Phys. Lett..

[53] Karpov SY (1998). MRS. Internet J. Nitride Semicond. Res..

[54] Ho HP, Lo KC, Siu GG, Surya C, Li KF, Cheah KW (2003). Mater. Chem. Phys..

[55] Chen WH, Kang XN, Hu XD, Lee R, Wang YJ, Yu TJ, Yang ZJ, Zhang GY, Shan L, Liu KX, Shan XD, You LP, Yu DP (2007). Appl. Phys. Lett..

[56] Cheng JH, Wu YS, Peng WC, Ouyang H (2009). J. Electrochem. Soc..

[57] Marks MR, Hassan Z, Cheong KY (2015). Crit. Rev. Solid State.

[58] Sheen M, Ko Y, Kim D-U, Kim J, Byun J-H, Choi Y, Ha J, Yeon KY, Kim D, Jung J, Choi J, Kim R, Yoo J, Kim I, Joo C, Hong N, Lee J, Jeon SH, Oh SH, Lee J, Ahn N, Lee C (2022). Nature.

[59] Wagner RS, Ellis WC (1964). Appl. Phys. Lett..

[60] Lieber CM (1998). Solid State Commun..

[61] Lauhon LJ, Gudiksen MS, Lieber CM (2004). Phil. Trans. R. Soc. Lond. A.

[62] Lu W, Lieber CM (2006). J. Phys. D Appl. Phys..

[63] Meng F, Morin SA, Forticaux A, Jin S (2013). Acc. Chem. Res..

[64] Noh S, Han S, Choi I, Kim JS, Ryu M-Y (2020). J. Kor. Phys. Soc..

[65] Mohammad SN (2006). J. Chem. Phys..

[66] Mohammad SN (2007). J. Chem. Phys..

[67] Hong YJ, Yoo J, Doh Y-J, Kang SH, Kong K-J, Kim M, Lee DR, Oh KH, Yi G-C (2009). J. Mater. Chem..

[68] Tang T-Y, Shiao W-Y, Lin C-H, Shen K-C, Huang J-J, Ting S-Y, Liu T-C, Yang C, Yao C-L, Yeh J-H (2009). J. Appl. Phys..

[69] Lin YT, Yeh TW, Nakajima Y, Dapkus PD (2014). Adv. Funct. Mater..

[70] Hong YJ, An SJ, Jung HS, Lee CH, Yi GC (2007). Adv. Mater..

[71] Ra Y-H, Wang R, Woo SY, Djavid M, Sadaf SM, Lee J, Botton GA, Mi Z (2016). Nano Lett..

[72] Kishino K, Sekiguchi H, Kikuchi A (2009). J. Cryst. Growth.

[73] Jung BO, Bae S-Y, Kato Y, Imura M, Lee D-S, Honda Y, Amano H (2014). Cryst. Eng. Comm..

[74] Choi K, Arita M, Arakawa Y (2012). J. Cryst. Growth.

[75] Guo W, Zhang M, Banerjee A, Bhattacharya P (2010). Nano Lett..

[76] Kim H, Farrell AC, Senanayake P, Lee W-J, Huffaker DL (2016). Nano Lett..

[77] Dede D, Glas F, Piazza V, Morgan N, Friedl M, Güniat L, Nur Dayi E, Balgarkashi A, Dubrovskii VG, A. Fontcuberta i Morral. (2022). Nanotechnology.

[78] Hasan SMN, You W, Ghosh A, Sadaf SM, Arafin S (2023). Cryst. Growth & Design.

[79] Alaskar Y, Arafin S, Wickramaratne D, Zurbuchen MA, He L, McKay J, Lin Q, Goorsky MS, Lake RK, Wang KL (2014). Adv. Funct. Mater..

[80] Koma A (1992). Thin Solid Films.

[81] Hong YJ, Lee WH, Wu Y, Ruoff RS, Fukui T (2012). Nano Lett..

[82] Hong YJ, Yang JW, Lee WH, Ruoff RS, Kim KS, Fukui T (2013). Adv. Mater..

[83] Wu C, Das Sarma S (2008). Phys. Rev. B.

[84] Kim Y, Cruz SS, Lee K, Alawode BO, Choi C, Song Y, Johnson JM, Heidelberger C, Kong W, Choi S, Qiao K, Almansouri I, Fitzgerald EA, Kong J, Kolpak AM, Hwang J, Kim J (2017). Nature.

[85] Ji J, Kwak H-M, Yu J, Park S, Park J-H, Kim H, Kim S, Kim S, Lee D-S, Kum HS (2023). Nano Convergence.

[86] Kong W, Li HS, Qiao K, Kim Y, Lee K, Nie YF, Lee D, Osadchy T, Molnar RJ, Gaskill DK, Myers-Ward RL, Daniels KM, Zhang YW, Sundram S, Yu Y, Bae SH, Rajan S, Shao-Horn Y, Cho K, Ougazzaden A, Grossman JC, Kim J (2018). Nat. Mater..

[87] Jeong J, Wang QX, Cha J, Jin DK, Shin DH, Kwon S, Kang BK, Jang JH, Yang WS, Choi YS, Yoo J, Kim JK, Lee CH, Lee S, Zakhidov AA, Hong S, Kim MJ, Hong YJ (2020). Sci. Adv..

[88] Li T, Guo W, Ma L, Li W, Yu Z, Han Z, Gao S, Liu L, Fan D, Wang Z, Yang Y, Lin W, Luo Z, Chen X, Dai N, Tu X, Pan D, Yao Y, Wang P, Nie Y, Wang J, Shi Y, Wang X (2021). Nat. Nanotechnol..

[89] Li F, Feng Y, Li Z, Ma C, Qu J, Wu X, Li D, Zhang X, Yang T, He Y, Li H, Hu X, Fan P, Chen Y, Zheng B, Zhu X, Wang X, Duan X, Pan A (2019). Adv. Mater..

[90] Luo R, Xu WW, Zhang Y, Wang Z, Wang X, Gao Y, Liu P, Chen M (2020). Nat. Commun..

[91] Mohseni PK, Behnam A, Wood JD, English CD, Lyding JW, Pop E, Li X (2013). Nano Lett..

[92] Kim H, Kim JC, Jeong Y, Yu J, Lu K, Lee D, Kim N, Jeong HY, Kim J, Kim S (2021). J. Appl. Phys..

[93] Roh I, Goh SH, Meng Y, Kim JS, Han S, Xu Z, Lee HE, Kim Y, Bae S-H (2023). Nano Convergence.

[94] Shemella P, Nayak SK (2009). Appl. Phys. Lett..

[95] J. Lyding, K. Ritter, K. He, J. Koepke, S. Schmucker, J. Wood, Y. Xu and N. Aluru. ECS Meeting Abstracts. MA2010–01 (2010)

[96] Xu Y, He KT, Schmucker SW, Guo Z, Koepke JC, Wood JD, Lyding JW, Aluru NR (2011). Nano Lett..

[97] Chae S, Jang S, Choi WJ, Kim YS, Chang H, Lee TI, Lee J-O (2017). Nano Lett..

[98] Kobayashi Y, Kumakura K, Akasaka T, Makimoto T (2012). Nature.

[99] Yoo J, Ahmed T, Chen R, Chen A, Kim YH, Kwon KC, Park CW, Kang HS, Jang HW, Hong YJ, Yang WS, Lee C-H (2018). Nanoscale.

[100] Wang X, Kim Y, Baldwin JK, Jones AC, Jeong J, Kang KT, Chen A, Yoo J (2021). J. Appl. Phys..

[101] Kim Y, Watt J, Ma XD, Ahmed T, Kim S, Kang K, Luk TS, Hong YJ, Yoo J (2022). ACS Nano.

[102] Wang Y, Qu Y, Xu Y, Li D, Lu Z, Li J, Su X, Wang G, Shi L, Zeng X, Wang J, Cao B, Xu K (2023). ACS Nano.

